# A Convolutional Neural Network Combining Discriminative Dictionary Learning and Sequence Tracking for Left Ventricular Detection

**DOI:** 10.3390/s21113693

**Published:** 2021-05-26

**Authors:** Xuchu Wang, Fusheng Wang, Yanmin Niu

**Affiliations:** 1 Key Laboratory of Optoelectronic Technology and Systems of Ministry of Education, College of Optoelectronic Engineering, Chongqing University, Chongqing 400044, China; 201808131090@cqu.edu.cn; 2 College of Computer and Information Science, Chongqing Normal University, Chongqing 400050, China; niuym@cqnu.edu.cn

**Keywords:** left ventricular detection, superpixel segmentation, scale adaptive anchors, discriminative dictionary learning, correlation filtering, convolutional neural network

## Abstract

Cardiac MRI left ventricular (LV) detection is frequently employed to assist cardiac registration or segmentation in computer-aided diagnosis of heart diseases. Focusing on the challenging problems in LV detection, such as the large span and varying size of LV areas in MRI, as well as the heterogeneous myocardial and blood pool parts in LV areas, a convolutional neural network (CNN) detection method combining discriminative dictionary learning and sequence tracking is proposed in this paper. To efficiently represent the different sub-objects in LV area, the method deploys discriminant dictionary to classify the superpixel oversegmented regions, then the target LV region is constructed by label merging and multi-scale adaptive anchors are generated in the target region for handling the varying sizes. Combining with non-differential anchors in regional proposal network, the left ventricle object is localized by the CNN based regression and classification strategy. In order to solve the problem of slow classification speed of discriminative dictionary, a fast generation module of left ventricular scale adaptive anchors based on sequence tracking is also proposed on the same individual. The method and its variants were tested on the heart atlas data set. Experimental results verified the effectiveness of the proposed method and according to some evaluation indicators, it obtained 92.95% in AP50 metric and it was the most competitive result compared to typical related methods. The combination of discriminative dictionary learning and scale adaptive anchor improves adaptability of the proposed algorithm to the varying left ventricular areas. This study would be beneficial in some cardiac image processing such as region-of-interest cropping and left ventricle volume measurement.

## 1. Introduction

Anatomical or tissue object detection has receiving increasing attention in medical image analysis field since it facilitates to isolate the area of interest from the background and determine the relevant position and category in medical images [1,2,3]. In the image-based assisted diagnosis process of cardiovascular diseases, it is an important preparation stage in the detection of left ventricle and myocardium in cardiovascular magnetic resonance imaging (MRI) slices. Accurate automatic left ventricular localization will provide great help for subsequent registration, segmentation, automatic ejection fraction calculation, and other tasks, so as to assist doctors in clinical diagnosis of heart health.

So far, a limited number of automatic cardiac object detection methods have been proposed [4,5] and some deep learning based methods have also been investigated [6,7]. However, it is a very challenging problem because of the complex distribution of various organs and tissues in cardiac MRI, intensity inhomogeneity, insignificant intensity differences between the myocardium and surrounding tissues, and large changes in the shape and size of the left ventricle region at different slice locations.

In this paper, we propose a convolutional neural network (CNN) method specifically designed to detect the left ventricle object in cardiac MRI slices. Our approach takes the detection into coarse oversegmentation location, discriminative dictinary learning-based location adjustment, and fine CNN-based detection parts with interpretability. The unsupervised SLIC (simple linear iterative cluster) algorithm is introduced to obtain initial oversegmentation because it has compact structure, good edge fitness, low complexity, and flexible adjustment. In addition, it does not require a preliminary training process in comparison to the CNN-based region proposal subnetwork. The discriminative dictionary learning can efficiently represent the different sub-objects in left ventricle area, and the sequence tracking fast generates left ventricular scale adaptive anchors on the same individual to solve the problem of slow classification speed of discriminative dictionary. Once multi-scale adaptive anchors are generated in the target region for handling the varying sizes, they are trained in regional proposal network, and the left ventricle object is localized by the CNN based regression and classification strategy. In the following sections we will present related works, our motivation, the details of our method, and experimental validation.

## 2. Related Works and Motivation

### 2.1. Related Works

Traditionally, the object detection methods in the computer vision field can be directly applied for left ventricular detection. The representative method was originally developed for face detection, which introduces a sliding window to traverse the entire image, designs single or multiple handcrafted features (scale invariant feature transform-SIFT [8], histograms of oriented gradients-HOG [9], etc.) to characterize the image area and builds traditional classification algorithms (support vector machines-SVM [10], AdaBoost [11], etc.) to classify the region. The semi-automatic approach [12] can even significantly reduce clinicians’ the amount of work. One obvious shortcoming in these methods arises from the redundant regional anchors since sliding windows are generated in an untargeted manner and another comes from the handcrafted features that have weak ability to represent the image region, which seriously affects the detection speed and performance.

To overcome the above limitations, many CNN (Convolutional Neural Network) based detection methods have been investigated and they achieved more satisfactory performance in nature images, these methods can be mainly divided into one-phase based, two-phase based, and anchor-free based methods.

One-stage object detection methods: This category aims to directly classify and regress the sliding windows, such as Yolo (you only look once) series [13,14,15], SSD (single shot multibox detector) [16], and their improvements. In the essential flowchart of this category, the input image is divided into multiple grids and each grid is responsible for predicting the target whose center falls within the grid and returns to the position and category of the bounding box directly at the output layer. Yolov3 is also used as automatic bounding box annotation in a recently proposed CPGGANs (Conditional Progressive Growing of Generative Adversarial Networks) to generate highly-rough bounding box conditions to place brain metastases at desired positions/sizes for MRI brain tumor detection [17]. It is characterized by fast detection speed but also the imbalance of positive and negative sample categories during training, resulting in poor robustness of targets with large morphological changes. To handle this, Retinanet [18] is proposed with a focal loss function to effectively alleviate category imbalances during training and achieved certain effects. On the other hand, Refinedet [19] is introduced to combine feature fusion and box regression from coarse to fine stages by following the SSD network structure, which improves certain accuracy and retains considerable detection speed.

Two-stage object detection methods: This category intends to generate proposals separately and then perform regression and classification. Representative algorithms include RCNN (regional CNN) [20], Fast RCNN [21], Faster RCNN [22], and their improvements. Although these algorithms are characterized by poor real-time performance, they have certain advantages in detection accuracy, and the intermediate process has strong interpretability. For example, the selective search technique [23] in RCNN generates region proposals with visible sense and is helpful for extracting CNN features one by one to feed classification and border regression. Fast RCNN introduces the idea of SPP-Net [24] to perform feature extraction on the entire image, achieving feature map sharing to improve the detection speed. Furthermore, Faster RCNN [22] uses RPN to generate region proposals and achieve end-to-end detection, which improves both detection speed and accuracy. In addition, R-FCN [25] removes all fully connected layers in Fast RCNN and proposes position sensitive score maps. Mask RCNN [26] presented a segmentation branch by using FCN (fully connected network) [27] for semantic segmentation, and it achieves the decoupling of mask and class prediction relationship where the introduced RoI Align instead of RoI pooling significantly improves the accuracy of the mask. The two-stage Mask-RCNN method is also proposed to detect and segment the optic nerve head and optic disc of retinal images [28], where the first stage aims to coarse positioning of the optic nerve head and the cropped images are used as the input of the second stage. The final result is largely dependent on the coarse positioning of the first stage. Recently, a cascade RCNN [29] is proposed to set different IOU (intersection over union) thresholds for positive and negative samples to improve the detection accuracy through multiple regressions on the bounding box. The cascaded localization regression network is also introduced for kidney localization [30], which detects the positions of kidney from two-dimensional cross-sectional slices in three orthogonal directions in one stage. The optimized training mechanism is also employed to improve the segmentation of left and right ventricles and myocardium on small sample data sets [31].

Anchor-free methods: This category is to directly predict the position of objects by inferring key point locations, such as Cornernet [32] and Centernet [33]. The idea of this category is to detect key points instead to key regions and has been applied in human posture estimation. To estimate a very large but extremely sparse bounding box dependent probability distribution, Denet [34] is proposed to extract a corner area of interest to filter most proposals by directed sparse sampling, and then employ it in a single end-to-end CNN based detection model. To alleviate the anchor box optimization in single shot detection methods, FSAF (feature select anchor-free module) [35] is proposed to combine the anchor-based and anchor-free branches, which includes anchor-free branch and online feature selection; the former is used for each pixel classification and coordinate regression, and the latter is responsible for online detection of each ground truth feature map. In addition, synthesized training data by generating adversarial networks can expand the lack of data in the real image distribution, balance the number of real and synthetic training data, and achieve the better detection performance with additional workload [36]. The quantification results by deep learning in multicenter study [37] show that CNN can reach the same accuracy as the expert measurement with fast quantification.

There are less reports of traditional methods on the detection of left ventricle in cardiac MRI. Focusing more on the location of left ventricle [4], automatic segmentation of left ventricular myocardial area can be smoothly employed [38,39]. In recent years, several studies have been published on left ventricular detection in MRI based on deep learning. The long short-term memory (LSTM) was proposed to detect left ventricular sequences [5], but the cardiac MRI sequence is not as coherent as the video sequence; on the contrary, some frames have mutation, and the network structure did not include a regression module, resulting in inaccurate detection frames. The sliding window strategy is also adopted to generate fixed size and fixed position proposals, and then CNN is used for classification [40]. Due to the fixed size and position of the proposals, the overlap rate between the detection box and ground-truth one is low. The unsupervised stacked sparse auto-encoders (SSAE) depth features was integrated into candidate region generation and SVM classifier and regressor were trained [7]. Although SSAE can learn better features, it can not effectively fuse myocardial and blood pool regions with significant gray-scale differences, thus affecting the accuracy of detection box. Besides this limitation, the number of positive samples is insufficient and there is a lack of diversity. At the same time, an excessive amount of negative samples leads to an imbalance of positive and negative samples in network training, and the detection results are not satisfactory. Recently, a deep learning-based left ventricle detection and classification method was proposed [6]; in this method, the additional convolution filter layer was added to enhance the feature map along with the dropout layer, which intrinsically belongs to the classification of blood pool and myocardium instead of the left ventricle object detection.

### 2.2. Motivation and Contribution

The design of many general frameworks in target detection does not depend on image categories. Although they can be transferred to medical MRI after modification, it is worth noting that medical MRI can only be obtained through professional medical equipment, unlike natural images, which can easily obtain a large number of data. In many cases, the natural images are sent to deep CNN such as Resnet152, Resnet101, or Resnet50 [41] for achieving good results. Due to the relatively small number of medical MRIs, it is often difficult to fit deeper network, so the shallow backbones are usually chosen In computer-assisted diagnosis and treatment, the accuracy counts more than the processing speed in most cases. At the same time, the interpretability and visualization of intermediate processes are important to assist the diagnosis of doctors. However, the one-stage detection algorithm and anchor-free algorithm often treat the network as a black box, and it is difficult to meet the practical application to obtain the detection results directly from the input images. Currently, the left ventricle detection is mainly based on the two-stage methods.

Based on the above considerations, this paper focuses on the two-stage convolutional network algorithms for left ventricle detection in cardiac MRI, where vgg-16 [42] is taken as the backbone.

**Figure 1:**
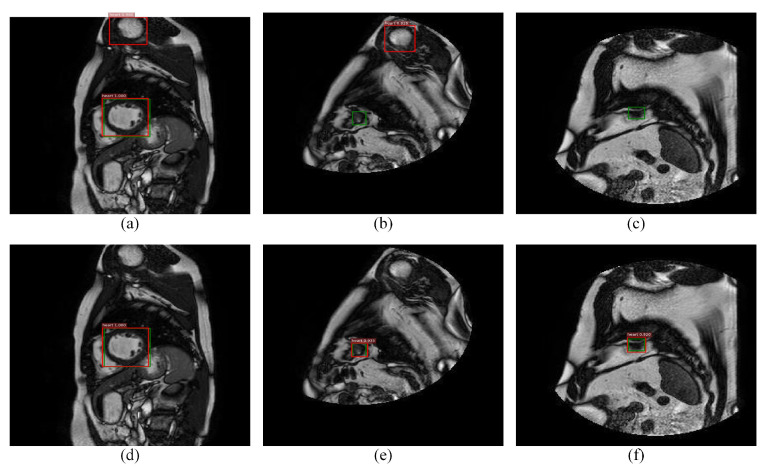
Detection examples of hard false positives and hard false negatives samples, where the first row (**a**–**c**) and the second row (**d**–**f**) are the results of Faster RCNN and the proposed method, where red and green denotes the detection rectangle and the ground truth in each image, respectively. It is seen that pseudo or small left ventricles easily lead to misdetection.

**Figure 2:**
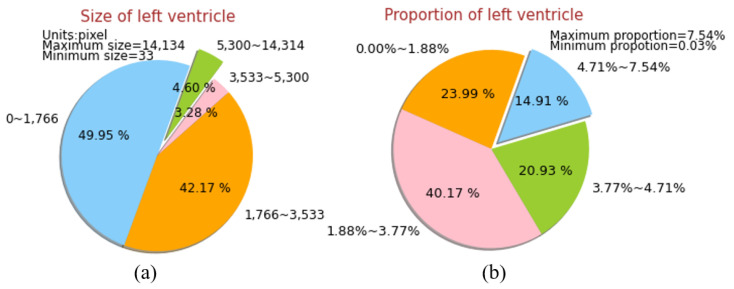
Varying sizes and small proportions of left ventricle area in the MRI data set. (**a**) Sizes of left ventricle area vary from 33 pixels to 14,134 pixels. (**b**) Proportions of the left ventricle area in the whole image vary from 0.03% to 7.54%.

Among the classical two-stage detection algorithms, Faster RCNN shows state-of-the-art performance. However, it has certain limitations when it is directly applied to cardiac MRI left ventricle detection. Figure 1 reports some examples of error detection and the possible reasons are mainly due to the following three points:

(1) The shape and size of the left ventricle vary greatly in cardiac MRI slices. As shown in Figure 2, the left ventricle target area has a large size span from 33 pixels to 14,134 pixels, and the size of the sliding anchors in the Faster RCNN is artificially set, the size of anchors is not significant and cannot be adapted to different left ventricular regions at different scales.

(2) The capture of perception area of RPN in Faster RCNN is weak. As shown in Figure 2, 49.96% of the size is below 1766 pixels, 23.99% of the left ventricle area accounts for less than 1.88% of the entire image, and part is a small size area. while the perception area of RPN in the original image is 16 × 16 (unit: pixel). In each slice, the anchor center may be far away from the center of the left ventricular area, the low overlap between anchor and ground-truth results in the imbalance of positive, and negative sample types in RPN training. At the same time, due to the lack of high-quality negative samples around the threshold boundary of positive and negative samples in the proposed layer, the network has poor discrimination ability for the hard to distinguish negative samples.

(3) The distribution of organs and tissues in cardiac MRI is complicated, and some organs and left ventricle have little difference. RPN generates non-differential anchors in the original image, which leads to misdetection of similar regions of the left ventricle. At the same time, due to the lack of adaptability of the generated anchors to small-sized ventricular regions, missed detection is prone to occur.

In view of the above problems, this paper proposes a CNN left ventricular detection algorithm combining discriminant dictionary learning and sequence tracking. The algorithm applies a discriminant dictionary learning to classify the superpixel oversegmented regions, then merge the target region of left ventricle, and generate multiscale adaptive anchors in the target region. Combining with non-differential anchors in RPN, the left ventricle is detected by CNN regression and classification strategy. In order to solve the problem of slow classification speed of discriminative dictionary, a fast generation module of left ventricular scale adaptive anchors based on sequence tracking is proposed on the same individual. Specifically, this paper provides three contributions as follows.

(1) During the training phase, this paper classifies the segmented superpixel regions by discriminative dictionary, fuses the same labels to construct the left ventricular candidate region, generates scale adaptive anchors in the candidate region, and combines anchors in RPN training to increase the number of positive samples and alleviate the imbalance problem of positive and negative samples, randomly perturb the scale adaptive anchors, construct the diversity of positive samples, obtain some high-quality negative samples, add them into the proposal layer to enhance the network’s discrimination of hard negative samples.

(2) During the detection phase, the adaptive anchors of the left ventricular region scale are mapped to RPN and proposal layers, respectively, to effectively prevent false detection and missed detection and enhance the robustness of the network to small-sized ventricular regions.

(3) Considering the slow classification speed of discriminative dictionary, this paper proposes a rapid generation module of scale adaptive anchors applicable to the left ventricle region in the same individual according to the idea in discriminative scale space tracking [43]. It can effectively improve the detection speed in the same individual.

The rest of this article is organized as follows. The second section presents the details of the proposed method, and the third section reports the experimental results and makes discussion. Finally, the fourth section concludes the study.

## 3. Method

### 3.1. Overview

**Figure 3:**
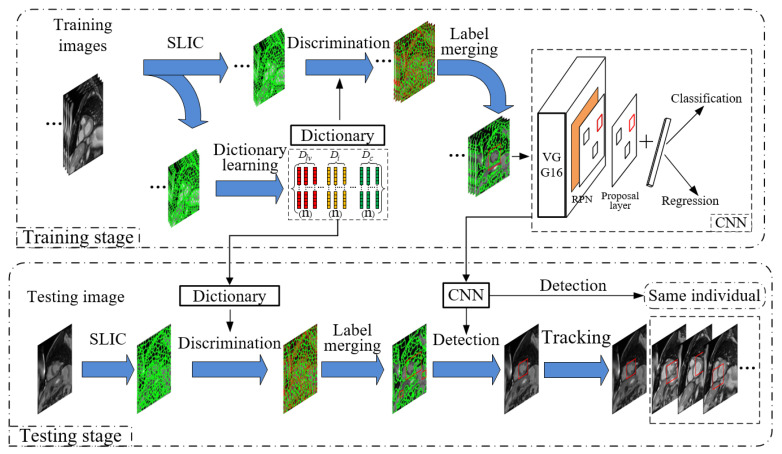
Overall framework of proposed left ventricular detection method, including modules of learning discriminative dictionary to classify superpixel regions, constructing left ventricular candidate regions through label fusion, generating scale adaptive anchors in candidate regions, and mapping to RPN and proposal layers.

The proposed overall framework of left ventricular detection in cardiac MRI is shown in Figure 3. It mainly consists of three modules:

(1) Left ventricle candidate region building module. This module adopts the unsupervised SLIC algorithm [44] to segment multiple MRI slices with left ventricular tags, clusters other regions outside the ventricle, and divides them into seven types of superpixel regions. The dictionary is trained from these superpixel regions and then applied in oversegmented testing images, where the superpixel regions are classified one by one, and a region fusion algorithm is performed by the same label to generate candidate regions of the left ventricle.

(2) Detection network module. This module follows the network structure of Faster RCNN, and uses vgg-16 to extract features. In the training phase, the left ventricular candidate region scale adaptive anchors are added to the RPN, and the diversity of positive samples is constructed by randomly perturbing the scale adaptive anchors, while generating some high-quality negative samples to be added to the proposal layer. During the test phase, anchors are mapped to the RPN and the proposal layer.

(3) Fast generation module of left ventricular scale adaptive anchors. According to the detection results of the current frame, all MRI slices of the same individual are stacked along the short axis. The module can quickly generate left ventricular scale adaptive anchors on each cardiac MRI along the frame, which significantly saves time cost and achieves high accuracy.

The details of the modules will be presented as follows.

### 3.2. Left Ventricular Candidate Region Generation Module

**Figure 4:**
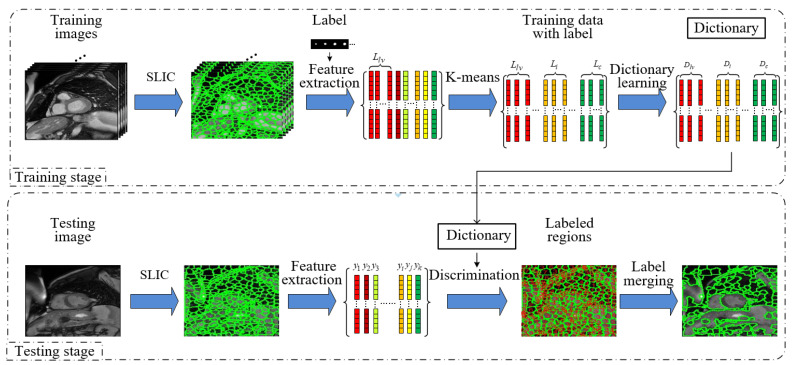
Block diagram of candidate region generation module. In the training stage, the seven sub-dictionaries are trained by seven types superpixel regions. In the testing stage, the discriminative dictionary is proposed to classify the segmented superpixel regions and fuse the same label areas to generate left ventricular candidate regions.

This module aims to automatically build the region candidates containing left ventricles based on oversegmentation and region merging techniques. As shown in Figure 4, the module is mainly divided into a training stage and a testing stage as below.

#### 3.2.1. Initial Oversegmentation

The initial oversegmentation is to generate a pile of superpixel candidates fast in an explainable way by borrowing the merits of the structural tightness and edge fitness of the cardiac images.

Due to the imperfection of magnetic resonance equipment, cardiac MRI is prone to artifacts and noise. In the preprocessing stage, the SLIC superpixel segmentation algorithm utilizes the redundant information between pixels and the effects of artifacts and noise can be eliminated through feature similarity. We use unsupervised SLIC oversegmentation to obtain initial superpixels because it has compact structure, good edge fitness, and flexible adjustment. In addition, the algorithm has low complexity and does not require a preliminary training process. Nevertheless, it is necessary to fuse them since the structural organization is significantly different and the superpixels do not cover the true left ventricle region exactly. Furthermore, according to the results of K-means clustering, the superpixel regions can be coarsely divided into six types related to left ventricle regions and one to background region. In this way, these seven types of regions will be further classified to their desired labels by learning strategies.

#### 3.2.2. Discriminative Dictionary Training

The oversegmented regions usually differ from the true regions due to the large gray discrepancy between the myocardium and the blood pool as well as the intensity inhomogeneity in the surrounding tissues. To handle this, we propose a discriminative dictionary learning method to fuse the myocardium and blood pool into the same region and then improve the accuracy of scale adaptive anchors generation which mainly depends on the results of these oversegmented regions. Traditional dictionary learning method is not suitable for this problem since it lacks discriminative power for each type of data. If the dictionary specific to each kind of data is learned, the sub-dictionaries are able to represent the data with same properties (i.e., blood pools or myocardium), while their representation ability for other kinds is poor. However, the nearby superpixels in the sub-dictionaries are usually overlapped to represent the partial object and when the left ventricle label is incorporated to enhance the class information in advance, the myocardium and blood pool will be taken into a same type for training a certain type of sub-dictionary in the dictionary, so it becomes possible to put both into one category in testing stage.

The discriminative dictionary learning can explicitly constrain the type of atoms in the dictionary to build a discriminative sub-dictionary [45], so we use it to increase the model’s ability by learning a dictionary with strong reconstruction and discriminating capabilities. Specifically, the objective of our model is

(1) $$\langle D,X\rangle =\arg\min_{D,X}\left\{I\left(Y,D,X\right)+\epsilon_{1}{\left||X|\right|}_{1}+\epsilon_{2}f\left(X\right)\right\}$$

where I(Y,D,X) is the reconstruction term that aims to increases the dictionary’s ability to identify each type of data during the reconstruction; f(X) is the function of sparse coding that can enhance the discrimination of sparse coding for each type of superpixel region. $\epsilon_{1}$ and $\epsilon_{2}$ are constants to adjust the weight of two parts. Furthermore, the definition of discriminative dictionary model is defined as

(2) I(yi,D,X)=||yi−DXi||F2+||yi−DiXii||F2+∑j=1j≠ik||DjXij||F2

where $D=\left[D_{1},D_{2},\cdots D_{c}\right]$ is the learned dictionary, $D_{i}$ is the *i*-th sub-dictionary of *D*, which corresponds to a certain type of superpixel region, denoting the superpixel region, $X_{i}=\left[X_{i}^{1},X_{i}^{2},\cdots X_{i}^{k}\right]$ is a sparse encoding $y_{i}$ on *D*, and $X_{i}^{i}$ is the sparse encoding of the *i*-th sub-dictionary of $X_{i}$. There are three constraint terms on the right side of the equation, where $\left|\right|y_{i}-DX_{i}{\left|\right|}_{F}^{2}$ is the reconstruction error. In the identification, it is necessary to ensure that the reconstruction error is sufficiently small. $\left|\right|y_{i}-D_{i}X_{i}^{i}{\left|\right|}_{F}^{2}$ is the discrimination term that tends to minimize the error between $y_{i}$ and a sub-dictionary $D_{i}$ and to identify the class most similar to the dictionary. $\sum_{j=1,j\neq i}^{k}\left|\right|D_{j}X_{i}^{j}{\left|\right|}_{F}^{2}$ is the constraint term of the remaining sub-dictionaries, which can make the *F*-norm of the remaining sub-dictionaries as small as possible.

The discriminant sparse coding term *f* is defined as

(3) $$f\left(X\right)=tr\left(S_{W}\left(X\right)\right)-tr\left(S_{B}\left(X\right)\right)+{∥X∥}_{F}^{2},$$

where $S_{w}\left(X\right)$ and $S_{B}\left(X\right)$ are the intra-class scatter and the inter-class scatter matrices, respectively, i.e., $S_{w}\left(X\right)=\sum_{i=1}^{c}{\sum_{x_{k}\in X_{i}}\left(x_{k}-m_{i}\right)\left(x_{k}-m_{i}\right)}^{T}$ and $S_{B}\left(X\right)=\sum_{i=1}^{c}n_{i}\left(m_{i}-m\right){\left(m_{i}-m\right)}^{T}$. *m* and $m_{i}$ are the mean of total sample and samples in the *i*-th class, respectively. $n_{i}$ is the number of training samples in the *i*-th class. By making the intra-class error as small as possible and the inter-class error as large as possible in the sparse coding, f(X) can enhance the ability of model discrimination.

So Equation (1) can be transformed as follows,

(4) $$\langle D,X\rangle =\arg\min_{D,X}\left\{I\left(y_{i},D,X_{i}\right)+\epsilon_{1}\left|\right|X_{i}{\left|\right|}_{1}+\epsilon_{2}f\left(X_{i}\right)\right\}.$$

The solution of the model is divided into two parts. $D(D=\left[D_{1},D_{2},\cdots D_{c}\right])$ is firstly fixed and $X(X=\left[X_{1},X_{2},\cdots X_{c}\right])$ is updated, then $D_{i}$ and $X_{i}$ are updated class-wisely during the solution. The weighting parameters $\epsilon_{1}$ and $\epsilon_{2}$ are settled as 0.1 and 0.001 respectively. Specifically, when *D* is fixed, *X* can be updated by using the IMP algorithm [46], that is,

(5) $$X_{i}=\arg\min_{X_{i}}\left\{I\left(y_{i},D,X_{i}\right)+\epsilon_{1}\left|\right|X_{i}{\left|\right|}_{1}+\epsilon_{2}f\left(X_{i}\right)\right\}.$$

It should be noted the remaining $X_{j}\left(j\neq i\right)$ are all fixed when $X_{i}$ is calculated. The above formula can be simplified as

(6) $$f_{i}\left(X_{i}\right)=\left|\right|X_{i}-m_{i}{\left|\right|}_{F}^{2}-\sum_{k=1}^{c}\left|\right|m_{k}-{m||}_{F}^{2}+\left|\right|X_{i}{\left|\right|}_{F}^{2},$$

where *m* and $m_{k}$ are the means of all classes and the *k*-th class of sparse coding, respectively. When *X* is fixed, then $D_{i}$ is updated class by class as follows,

(7) $$D_{i}=\arg\min_{D_{i}}\left\{\begin{array}{c} ||Y-D_{i}X^{i}-\sum_{j=1,j\neq i}^{c}D_{j}X^{j}{\left|\right|}_{F}^{2}\\ +\left|\right|y_{i}-D_{i}X_{i}^{i}{\left|\right|}_{F}^{2}+\sum_{j=1,j\neq i}^{c}\left|\right|D_{i}X_{j}^{i}{\left|\right|}_{F}^{2} \end{array}\right\},$$

where $X^{i}$ is the sparse encoding of the entire training data set *Y* on $D_{i}$. When $D_{i}$ is calculated, the remaining $D_{j}\left(j\neq i\right)$ are all fixed, and they are updated in the following steps.

(1) Initialize dictionary *D*. In each type of sub-dictionary $D_{i}\left(\;i=1,2\cdots c\right)$, randomly select *n* atoms as c×n initialization atoms of *D*, and there are totally n×c atoms.

(2) Fix *D*, update the sparse coding $X=\left[X_{1},X_{2},\cdots X_{c}\right]$, and calculate it by Equation (6).

(3) Fix *X*, update dictionary D, and calculate it by using Equation (7).

(4) Return to step 2 and iteratively update until the values of *D* and *X* in two adjacent iterations are close enough, or until the maximum number of iterations, then output *X* and *D*.

#### 3.2.3. Dictionary Testing

In the testing stage, a single class of sub-dictionary is used to reconstruct a certain superpixel region *y* and excludes the influence of the remaining sub-dictionaries. Let β bd a sparse encoding of *y* on dictionary *D* and it is solved as follows

(8) $$\beta =\arg\min_{\beta }\left\{||y-D_{i}\beta ||+\eta_{1}{\left||\beta |\right|}_{1}+\eta_{2}||\beta -m_{i}^{i}{\left|\right|}_{2}^{2}\right\},$$

where $\eta_{1}$ and $\eta_{2}$ are constants.

Furthermore, the label is estimated by combining reconstruction error and sparse coding error as follows,

(9) $$L_{i}=\arg\min_{i}\left\{||y-D_{i}{\beta ||}_{2}^{2}+\eta_{1}{\left||\beta |\right|}_{1}+\eta_{2}||\beta -m_{i}^{i}{\left|\right|}_{2}^{2}\right\},$$

where the smallest $L_{i}$ corresponds to the classified superpixel region. The weighting parameters $\eta_{1}$ and $\eta_{2}$ are settled as 0.1 and 0.005 respectively. Once the minimum $L_{i}$ is obtained, the superpixel region is assigned a label corresponding to the sub-dictionary $D_{i}$.

#### 3.2.4. Superpixel Region Fusion

Since initial superpixel region of each image is able to be labeled according to the discriminative dictionary learning, the objective of fusion strategy is to merge the regions with the same label. In the ideal case, the left ventricular region can be completed distinguished by discriminating the superpixel region of the ventricle through a discriminative dictionary, but in practice, due to the complex distribution of various tissues and organs in cardiac MRI images, as well as the subtle differences between some tissues and organs and the left ventricle, some organs that scatter in different positions in the image are easily misjudged as left ventricle, so in the following the detection network is described for accurate classification and localization.

**Figure 5:**
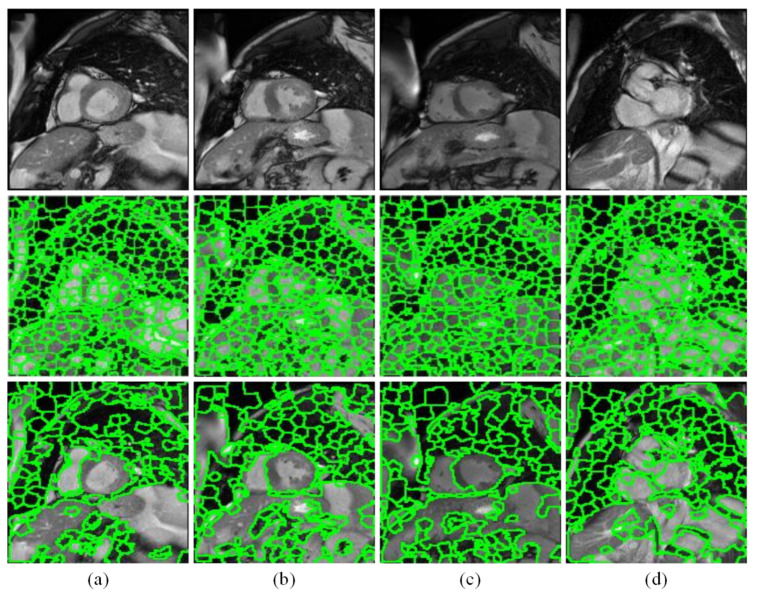
Candidate region generation results. (**a**–**d**) are four examples. From top to bottom are the original images, initial superpixel segmentation images, and proposed candidate region images.

Figure 5 shows some superpixel region fusion examples, from where it can be seen that in comparison to the results of initial SLIC oversegmentation, the left ventricular regions are more remarkable after the processing of proposed discriminative dictionary learning and label fusion. However, the results of regional fusion perform still not very well in some highly complicated images. So it is necessary to compensate them and correct the partially misjudged regions by designing the detection network with the scale adaptive anchors.

### 3.3. Detection Network and Setting of Scale Adaptive Anchors

The detection network in this paper adopts the regional proposal network (RPN) that is modified with scale adaptive anchors. In order to match the number of anchors slipped out of each point of the RPN, the scale adaptive anchors generated correspond to 9 anchors for each point. In the training phase, the Euclidean distances are computed between the center of the label and all the centers in the training images that are identified as left ventricular candidate regions, then the smallest distance is regarded as a significant left ventricular candidate region. In short-axis cardiac MRI images, the left ventricle region is close to a circle, so the precise bounding box should be close to a square. The minimum bounding box is generated in the salient left ventricle region in each training image, which is called the saliency anchor. If this saliency anchor is rectangular, a long and short side of the saliency anchor are used to generate two square enclosing boxes in the same center, which are three basic enclosing boxes. If the saliency anchor is square, the diagonal length and semi-diagonal length of the saliency anchor are used to generate two square enclosing boxes in the same center, which is the three basic bounding boxes. The length and width of the 3 basic bounding boxes are multiplied by the scale scaling factor $s_{1}$ and $s_{2}$ to generate 6 bounding boxes. The centers of the 9 scale adaptive anchors are mapped onto the feature map to replace the candidate anchors corresponding to the current point in the RPN. This setting approach will make the anchors have a good IOU value even if the regional fusion may produce some errors.

In order to further alleviate the shortage of positive samples, the sample augmentation was taken, where nine scale adaptive anchors’ centers were generated by randomly disturbance in the significance anchor and replacing the candidate boxes corresponding to the points around the current RPN points. In this way, the positive samples with diversity are constructed, and the high-quality negative samples around the threshold boundary are also built to join the proposal layer, so as to enhance the network’s ability to identify the hard to distinguish negative samples. In the testing stage, the scale adaptive anchors are mapped to the RPN and proposal layer on all left ventricular candidate regions for detection.

### 3.4. Left Ventricular Region Scale Adaptive Anchors Rapid Generation Module

**Figure 6:**
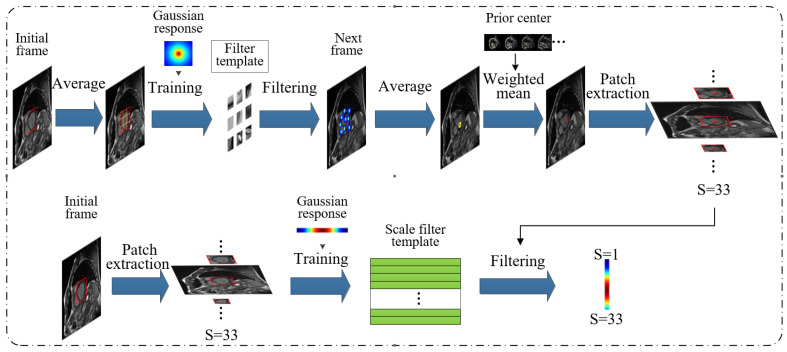
Fast-scale adaptive anchors generation module, where significant anchors are quickly generated through correlation filtering based on the current frame detection result.

The cardiac MRI slices contain a series of images of multiple individuals at different times. To speed up the detection, this paper proposes a rapid generation module of adaptive anchors suitable for left ventricular regional scale for the same individual. The overall framework is shown in Figure 6.

Due to the complex tissue distribution and low discrimination of various organs in medical MRI images, it is easy to make the estimated center position significantly different from the true center if the dictionary learning algorithm is directly applied. The scale adaptive anchors in this paper are generated in the same center; the accurate results are hard to obtain if the center position is not accurate. To handle this, the detection boxes are divided into 9 small blocks, and the estimated center positions of these blocks are averaged to obtain the prediction center. The final central location is computed as the weighting location of the prediction center and the prior center respectively. At the new center position, the left ventricular saliency anchor is obtained through a multiscale filter template, and the scale anchors are set on the saliency anchor in the same manner. Subsequent detection does not need to generate significant anchors through discriminative dictionary classification and label fusion, which effectively improves the detection speed and achieves competitive results. The training of template constructs the optimal correlation filter *h* by establishing a minimized cost function as follows

(10) $$\varepsilon =\left|\right|\sum_{l=1}^{d}h^{l}*f^{l}-g{\left|\right|}^{2}+\lambda \sum_{l=1}^{d}\left|\right|h^{l}{\left|\right|}^{2}$$

where *g* is Gaussian response and *l* represents a certain dimension of the feature. λ is a regular term coefficient for eliminating the influence of the zero frequency component in the *f* spectrum. It is empirically set as 0.01. This filter can be solved by a transformed into the frequency domain as follows,

(11) $$H_{t}=\frac{\bar{G}F^{l}}{\sum_{k=1}^{d}\bar{F^{k}}F^{k}+\lambda }$$

where *G* is the conjugate of the two-dimensional Gaussian response to the two-dimensional Fourier spectrum; $F^{k}$ is the two-dimensional Fourier spectrum of the *k*-th dimension of the current frame, $\bar{F^{k}}$ is the conjugate of the two-dimensional Fourier spectrum of the *k*-th dimension feature of the current frame, *H* is split into numerator *A* and denominator *B*, and it is iteratively updated as follows

(12) $$\begin{array}{c} A_{t}^{l}=\left(1-\theta \right)A_{t-1}^{l}+\theta \bar{G_{t}}F_{t}^{l}\\ B_{t}=\left(1-\theta \right)B_{t-1}+\theta \sum_{k=1}^{d}\bar{F_{t}^{k}}F_{t}^{k} \end{array}$$

where the learning rate θ is set as 0.025 and the target positions of 9 small blocks can be obtained by solving the maximum correlation filter response value as follows

(13) $$y=F^{-1}\left\{\frac{\sum_{l=1}^{d}\bar{A^{l}}Z^{l}}{B+\lambda }\right\}$$

In the above formula, $Z^{l}$ is the two-dimensional Fourier spectrum of the *k*-th dimension feature of the new frame. Once the new center point is estimated, candidate patches of different scales are obtained at the new center, and the best matching scale is obtained through the one-dimensional scale filtering template. The method of obtaining is similar to the position estimation, the choice of scale is shown as $a^{n}w\times a^{n}h;n\in \left\{\left[-\frac{s-1}{2}\right],\cdots \left[\frac{s-1}{2}\right]\right\}$, where *w* and *h* are the width and height of the previous frame, respectively, and s=33 is the number of scales.

The tracking of each individual is mainly divided into two steps: tracking of location and tracking of changes in scale. The flowchart of the main tracking algorithm is as follows:

(i) Location estimation.

(1) According to the detection position of the current frame, the detection region is averagely divided into 9 regions $Z_{trans}$ for training 9 $A_{t}^{trans}$ and $B_{t}^{trans}$.

(2) At the next frame, using 9 $Z_{trans}$ and 9 small blocks of $A_{t}^{trans}$ and $B_{t}^{trans}$ to calculate $y_{trans}$ respectively.

(3) Calculating $\max\left(y_{trans}^{1}\right)\cdots \max\left(y_{trans}^{9}\right)$ and averaging the center positions of the 9 small blocks to obtain the predicted center position $P_{1}$. The new center position $P_{t}$ of the target is obtained by weighting and averaging $P_{1}$ and the prior center $P_{2}$ with weight coefficients $w_{1}$ and $w_{2}$.

(ii) Scale estimation.

(1) At the new center of the target, extracting samples $Z_{trans}$ of 33 different scales.

(2) Calculating $y_{scale}$ by using $Z_{trans}$ and $A_{t}^{trans}$ and $B_{t}^{trans}$ of the previous frame.

(3) Calculating $\max(y_{scale})$ to get the accurate scale $S_{t}$ of the target.

(iii) Update.

Using the current frame information, the position template and the scale template are updated by the above update equations, where $A_{t}^{trans}=\bar{G}F^{l}$ and $B_{t}^{trans}=\sum_{k=1}^{d}\bar{F^{k}}F^{k}+\lambda$ are used to generate filtering templates in the process, also $Z_{trans}$ is the Fourier spectrum.

## 4. Data Sets and Evaluation Metrics

### 4.1. Cardiac Mri Data Set

The experimental data are from the cardiac atlas project (CAP) database. This data set was created by a collaborative database of multiple organizations [47], among which MRI acquisition equipment includes Siemens (Avanto 1.5T, Espree 1.5T, Symphony 1.5T), Philips (Achieva 1.5T, 3.0T, Intera 1.5T) and GE (Signa 1.5T), etc., due to different acquisition equipment, the image sequence is very different, the image size is 192 × 156 ∼ 512 × 512, and the voxel resolution is 0.7 × 0.7 × 6 ∼ 2.0 × 2.0 × 2.0 (unit: ${\mathrm{mm}}^{3}$). The data set contains a total of 83 patients with short-axis cardiac MRI. In one cardiac cycle, it contains about 18 to 35 volumes according to different time sampling intervals, and each volume contains about 8 to 17 images according to different space sampling interval along the short axis. Due to the imperfection of magnetic resonance equipment and the specificity of the subject, the MRI images inevitably present a certain degree of uneven brightness and intensity inhomogeneity. In our experiments, the bias field correction was performed on the image in the preprocessing stage, and the images with incomplete labels in the data set were eliminated. Totally, the experimental data set consists of 19,448 cardiac MRI images.

The myocardium region in each image was manually labeled by two well-trained students. Since this labeling was a non-trivial and challenging problem due to the fuzzy organ boundaries and large anatomical variability, their results were carefully cross-checked and further checked by a radiologist to made a final result as gold standard for evaluation. For each subject, this processing took about 2 h.

### 4.2. Evaluation Metrics

The detection performance of the model is comprehensively evaluated by the following measurements. Considering accuracy and recall measures usually received special attention in the medical image processing field, in our experiment the precision (P), recall (R), F1 score, AP50 and AP75 are commonly taken as evaluation indexes in our object detection tasks. In order to reflect the adaptability of the model to different individuals, 10 different individuals were randomly selected from the test set and evaluated using the AP50, AP75, and mAP indicators. In order to ensure the uniformity of the evaluation results, the threshold score of 0.7 in Faster RCNN is used in the experiment. The prediction confidence score is greater than 0.7 as the correct detection, otherwise as the false detection.

When calculating P and R, the indicators are defined as follows. The number of samples in which the left ventricular region is correctly predicted as the left ventricular region is TP (true positive). The number of samples in which the background region was incorrectly predicted as the left ventricular region was FP (false positive). The number of samples in which the background region is correctly predicted as the background region is TN (true negative). The number of samples in which the left ventricular region was incorrectly predicted as the background is FN (false negative). Based on them, accuracy represents the ratio of the number of positive samples that are correctly predicted to the ventricular area to the number of all divided positive samples, i.e.,

(14) $$P=\frac{TP}{TP+FP}$$

The recall rate is the ratio of the number of positive samples that are correctly predicted in the left ventricle area to the number of all true positive samples. i.e.,

(15) $$R=\frac{TP}{TP+FN}$$

F1 is the harmonic mean of the *P* and *R* measures. It can be used to measure the similarity of the two sets, i.e.,

(16) $$F1=\frac{2PR}{P+R}$$

The experiments were carried out on 2.0 GHz Intel CPU, 48GB RAM, NVIDIA RTX 2080Ti, Linux 64 bit PC. the programming environment was Anaconda 5.0.1 (Python 3.6), tensorflow1.4, keras2.0.8.

## 5. Experimental Results and Discussion

### 5.1. Parameter Settings

#### 5.1.1. Left Ventricle Candidate Region Building Module

**Figure 7:**
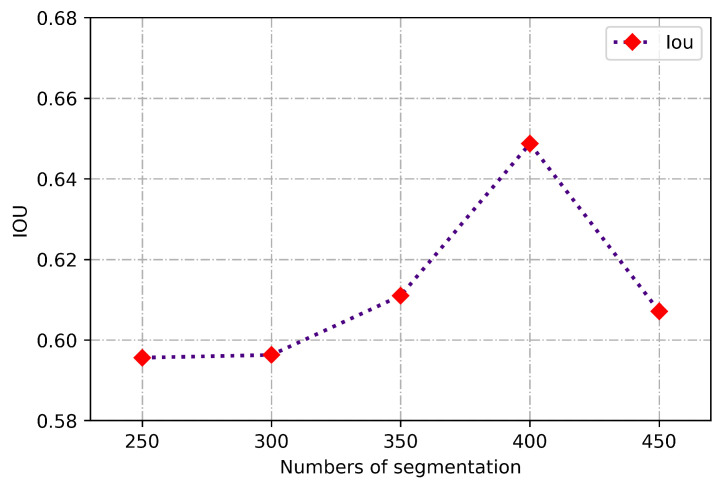
Curve of the merged IOU and the initial number of segmentation.

For the dictionary training data set, 10 different individual legends are randomly selected from the total training set, and a complete slice sequence is randomly selected from the 10 different legends to build the discriminatice dictionary. In the process of experiment, the weight coefficient follows the original model. Before training of discriminative dictionary learning, PCA is used to reduce the dimensionality of various training data and retain core atoms. The initial segmentation number in the experiment is set to *M*. If *M* is too large, the fitting degree of the edges of various tissues and organs will not be accurate, and the resulting IOU after the merging processing will be low and vice versa. In order to get optimal parameters, the relationship between the IOU after merging and the initial number of segmentation was experimentally investigated and the results are shown in Figure 7, from where it is seen that the IOU achieves the highest value when *M* is 400. Therefore, the number of initial superpixel is finally determined as 400.

#### 5.1.2. Detection Network Module

In the network parameter setting, the data augment approaches such as horizontal flip-up extended data set were used. The optimization approach was taken as the stochastic gradient descent method and the learning rate was set to 0.001 and the training batch was 256 anchors. According to the candidate box IOU as a positive and negative sample partitioning criterion, the RPN settled IOU > 0.7 as a positive sample, IOU < 0.3 as a negative sample, and the NMS [48] threshold was set to 0.7. In the proposal layer, IOU > 0.5 was set as a positive sample, and 0.1 < IOU < 0.5 was set as a negative sample. In order to adapt to the cardiac MRI data set, the size of the anchor was adjusted as 64 × 64, 128 × 128 and 256 × 256. The number of iterations was set to 50,000 and the weight decay rate was 0.0005.

**Figure 8:**
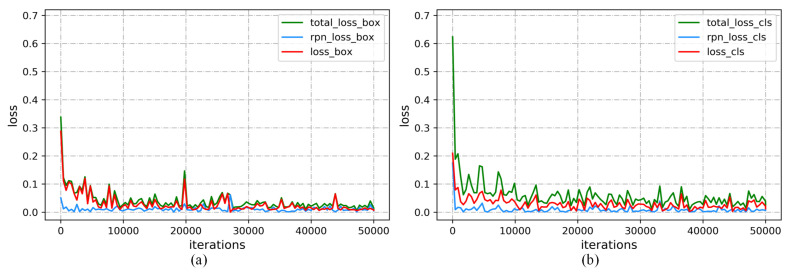
Training loss curves: (**a**) the regression loss and (**b**) the classified loss.

The training loss curves including regression part and classification part are shown in Figure 8, from where it can be seen that, with the increase of the number of iterations, each loss value gradually decreases, indicating that the model gradually adapts to the transformation of candidate regions and performs effective classification and regression adjustment on candidate anchors. However, there are some jumps in the curve. The main reason for the jumps is that there are larger errors between the significant left ventricular region and the real region in small parts of MRI; in addition, there are also some impacts rising from different image parameters collected by different devices.

#### 5.1.3. Saliency Anchors Fast Generation Module

**Figure 9:**
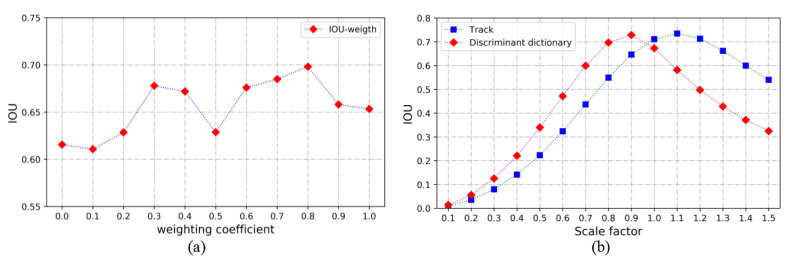
(**a**) Average IOU vs. the weight coefficient of the prior center. (**b**) Average IOU vs. the scale scaling factor.

In the left ventricular scale adaptive anchors rapid generation module in the experiments, λ was set to 0.01; the learning rate was set to 0.025; the scale factor was set to 1.02, and the number of scales was set to 33. In our experiment 20 slice sequences were randomly selected in the training set, and their label boxes were used as ground truth to explore the relationship between the prior center weight coefficient and the average IOU. The experimental results are shown in Figure 9a. It can be seen from the figure that the curve has small fluctuations and the average IOU is above 0.6, when the weight coefficients $w_{1}$ = 0.2 and $w_{2}$ = 0.8, the maximum average IOU can be obtained, so they are selected as the optimal parameters.

#### 5.1.4. Scale Adaptive Anchor Scaling Factor

The scale scaling factor in our model is a key parameter in generating scale adaptive anchors; if it is not set properly, the anchors will be difficult to fit different scale ventricular regions. The relationship between the average IOU and the scaling factor was explored by randomly selecting 5000 images in the training data set and then calculating the average IOU of the scale adaptive anchors under different scale factors. The results are shown in Figure 9b, where the IOUs of the two curves first increase and then decrease along with the increasing scale factor, and both reach their maximum values near the original basic frame (that is, the scale factor is 1). In sequence tracking generating scale adaptive anchors, the two scaling factors with the largest average IOU values are 1.1 and 1.2 respectively; in discriminant dictionary generating scale adaptive anchors, the two scale scaling factors with the largest average IOU values are respectively 0.8 and 0.9. So when the scale adaptive anchors fast generation module generates scale adaptive anchors, the scale scaling factor $s_{1}$ was set as 1.1 and $s_{2}$ was set as 1.2. Furthermore, when the discriminant dictionary generates scale adaptive anchors, the scale scaling factor $s_{1}$ was set as 1.1 and $s_{2}$ was set as 1.2.

### 5.2. Experimental Results

To thoroughly investigate the performance of the proposed method, three variant methods were built as follows. (1) Discriminant dictionary+Faster method. This variant one focuses on the effect of discriminant dictionary module, by combining it to the Faster RCNN; (2) Correlation filter + Faster method. This variant one focuses on the correlation tracking module; (3) Correlation filter + Proposal method. This variant one combines the left ventricular scale adaptive anchors generation module and the proposal layer to improve the detection speed. Furthermore, to more comprehensively evaluate the proposed algorithm, the state-of-the-art two-stage methods such as Faster RCNN [22], one-stage detection algorithms such as representative Retinanet [18], Yolov3 [14], SSD [16], and anchor-free method such as Centernet [33] are also introduced for comparison. The results of these comparative methods are shown in Table 1.

In order to explore the adaptability of the detection algorithm to different individuals, the model was tested on 10 different individuals randomly selected in the test set. Using the ground-truth images as the standard, the test results were evaluated using AP50, AP75, and mAP. The comparison results are reported in Table 2.

As can be seen from the above table, the model has different adaptability to different individuals. The proposed method and its variant can achieve significant performance improvements based on Faster RCNN on most individuals. In the evaluation of the mAP50 index, the method Correlation filter + Faster in this paper achieved competitive results in all comparison methods, which is 4.1% higher than Faster RCNN. In mAP75, the method and its variants outperforms 2.2∼3.6% improvement in comparison to Faster RCNN. but less than RetinaNet.

**Figure 10:**
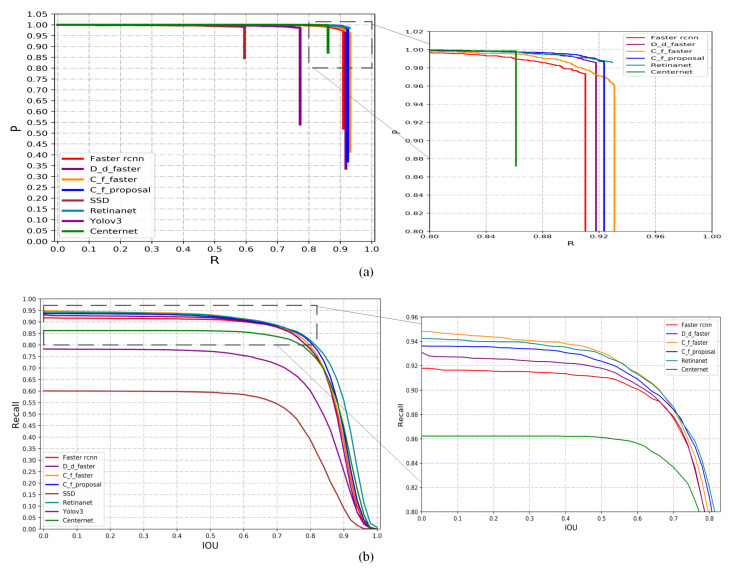
P-R curves (**a**) and recall-IOU curves (**b**) with zoomed parts of the proposed and compared methods and a series of detection methods. The proposed method shows competitiveness in all the zoomed curves.

Figure 10 plots the P-R curve of the proposed method and a series of classic detection algorithms on the testing set, as well as the recall-IOU curve. It can be seen from Figure 10a that the proposed method and variants in this paper are better than Faster RCNN. After the detection algorithm reaches a certain recall rate, the recall index will not change, and the detection accuracy will gradually decrease. The reason is that below a certain point, no left ventricular region has been recalled, and some error regions have been recalled. In the comparison of a series of classic detection algorithms, the method Correlation filter + Faster in this paper achieved competitive results. As can be seen from the above Figure 10b, the method and variants of this paper have better recall index than Faster RCNN. By adding scale adaptive anchors, it effectively prevents the missed detection of small scale left ventricular areas in Faster RCNN. Under the condition of IOU < 0.7 In this paper, the method Correlation filter + Faster will gradually surpass Retinanet and achieve competitive results on the recall rate index.

In order to compare the P-R curves of each method, the enlarged parts of the curves are plotted in the right column. It can be seen from the zoomed parts and the larger area under the PR curves that when IOU < 0.7, the proposed Correlation filter + Faster method achieves better performance than the compared methods.

**Figure 11:**
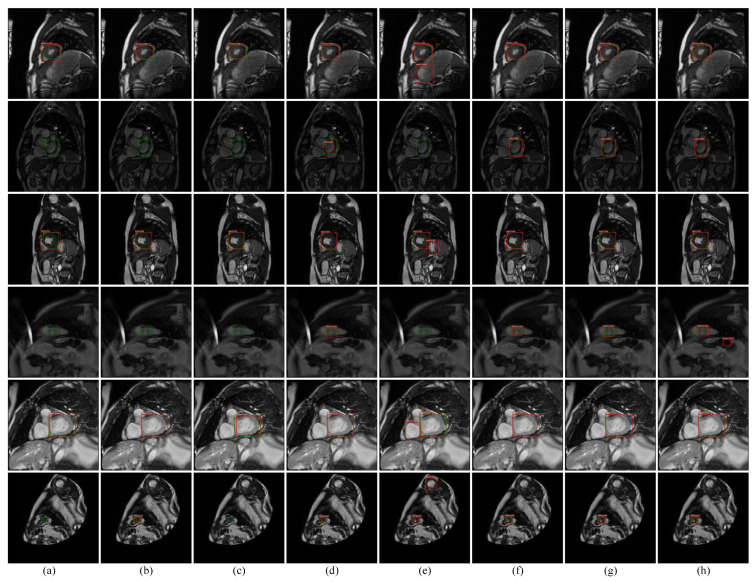
Detection examples by proposed methods and compared detection methods. (**a**–**h**) are SSD, Retinanet, Yolov3, Centernet, Faster RCNN, Correlation filter + Proposal, Discriminant dictionary + Faster, Correlation filter + Faster. The red box in the figure is the detection box, while the green box is the label box.

As can be seen from Figure 11, the method in this paper has achieved competitive results in comparison with the detection results of a series of methods, which can effectively avoid missed and false detections in Faster RCNN, Because Faster RCNN generates undifferentiated anchors on the entire map, when areas with similar shapes and appearances to the left ventricle are more likely to be misdetected, the anchors generated at the same time have poor robustness to small-scale regions, which leads to easy missed detection in images with smaller left ventricular regions. The left ventricular region scale adaptive anchors generated by the proposed method have adaptability to left ventricular regions of different scales, enhancing the significance of candidate anchors around the left ventricle region and improving the network’s ability to identify difficult negative samples.

**Figure 12:**
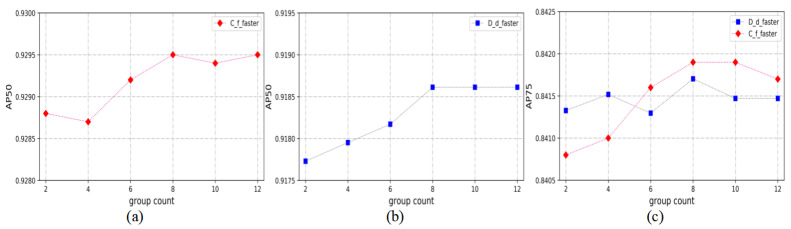
(**a**) is the graph of AP50 and the number of scale scaling factor groups in Correlation filter + Faster, (**b**) is the graph of AP50 and the number of scale factor groups in the Discriminant dictionary + Faster, (**c**) is the graph of AP75 and the number of scale factor groups in two methods.

In the detection stage, this paper explores whether the number of scale adaptive anchors is better. At the scale scaling factor of 0.4 to 1.5, two sets of scale adaptive anchors are added each time, and RPN and the proper layer are gradually added. After experiments were performed on the testing set, AP50 and AP75 were computed for evaluation by using the label box as the standard. The results are shown in Figure 12.

As can be seen from the figure, the AP50 and AP75 indicators have no significant relationship with the number of groups of scale scaling factors, and the indicators change within a small range as the number of groups changes.

### 5.3. Discussion

#### 5.3.1. Overall Performance

The proposed method and variants of this article have presented competitive performance in the experiments. As can be seen from the tables, they have been improved to varying degrees on the basis of Faster RCNN. In the comparison of the AP50 and AP75 indicators, the variant method Correlation filter + Faster achieved the most advanced results on the AP50 indicator, which increased by 2.1% on the basis of Faster RCNN. The variant method Correlation filter + Proposal of this paper has achieved comparable detection results with retinanet on the AP75 indicator and has increased by 1.3% on the basis of Faster RCNN. In terms of the recall rate index, the method and variants of this article are better than Faster RCNN. Under the condition of IOU = 0.5, the Correlation filter + Faster method has achieved a competitive result, which is 2% higher than Faster RCNN. Under the condition of 0.75, the variation method Correlation filter + Proposal in this paper achieved a detection performance comparable to Retinanet, which improved 1.2% on the basis of Faster RCNN. The experimental results show that the left ventricular scale adaptive anchors proposed in this paper can prevent the left ventricle area from being missed to some extent due to the adaptability to the left ventricular area of different scales.

In terms of accuracy index, the method Discriminant dictionary + Faster and the variant method Correlation filter + Proposal both achieved certain improvement on Faster RCNN. The main reason is that after the left ventricular scale adaptive anchors are added to the non-differential anchors in the RPN, the candidate boxes around the left ventricle region are more significant, making this part of the candidate boxes more prominent in the network during training and testing and in MRI; although there are regions similar to the left ventricular region, the surrounding candidate anchors are not significant, so the occurrence of false detection is suppressed to a certain extent. At the same time, it should be noted that the Correlation filter + Faster is lower than the Faster RCNN in the accuracy index, and the performance at IOU = 0.75 is lower than IOU = 0.5. The possible reasons are conjectured as follows. In the training, the scale adaptive anchors generated by dictionary learning are used. While in the detection, the scale adaptive anchors generated by tracking are used. The scale changes of these two anchors during the regression process and the change of the moving distance are different, which leads to the low IOU of the detection boxes. Some of the detection boxes were not included in the correct number of detection due to the low IOU, so that certain accuracy was lost.

#### 5.3.2. Time Cost

The proposed method was compared with a series of classic detection algorithms on the detection time index. During the experiment, the models were performed under the same experimental conditions as much as possible. The results are reported in Table 3.

As can be seen from the table above, in the one-stage algorithm, the detection speed of Yolov3 has achieved a leading advantage. The method of this paper, Discriminant dictionary + Faster and Correlation filter + Faster, has a higher time cost than Faster RCNN. In particular, in the Discriminant dictionary + Faster method, each time a discriminative dictionary classifies a superpixel region, iteration is needed, resulting in high time cost. After using the left ventricular region scale anchors to quickly generate the module, the time cost is significantly saved, and the variant Correlation filter + Proposal method, because the previous RPN and other convolutional layers are removed, directly cascades the left ventricular-scale adaptive anchors module with the proposal, effectively increasing the speed and gaining 7% on Faster RCNN.

#### 5.3.3. Limitation of Our Method

**Figure 13:**
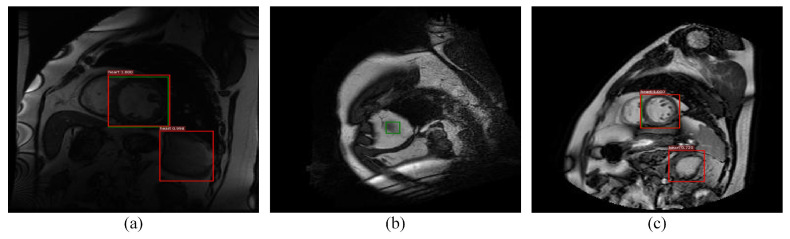
Examples of false or missed detection in the proposed method, where (**a**,**c**) are examples of false detection, and (**b**) is an example of missed detection. The green boxes and red ones denote the label and detection results, respectively.

The detection performance of the proposed method is significantly improved in comparison to Faster RCNN, which can effectively prevent false detection and missed detection. The comparison with a series of detection methods validates the effectiveness of the improved method in this paper. However, due to the complex tissue and organ distribution in MRI, the shape characteristics of some left ventricle areas are not obvious, and some areas are highly similar to the left ventricle; it is inevitable that a small amount of missed or false detection will occur. Figure 13 reports some examples of false or missed detection by the proposed method.

As can be seen from the figures above, the occurrence of pseudo-detection is mainly in areas where the appearance is similar to that of the left ventricle. Missing detection mainly occurs in the area where the the left ventricle shape features are not obvious and the size is too small. In further research, the adaptive and discriminant dictionary learning will be investigated for this challenging case.

It should be noted that this study treated 3D image volumes as 2D images for left ventricle detection in the training stage and testing stage. The 3D CNN was not applied to build the detect network mainly due to two facts. One was that the anisotropic property in the volumes makes 3D CNN hard to extract the feature along the short axis. Typically, the Z-interval is 6mm while the XOY spacing is 0.7mm in the data set. The other reason was based on the consideration of the training time of 3D CNN in comparison to 2D CNN.

Nevertheless, the structure of the proposed method cannot be called independent 2D CNN since a fast-scale adaptive anchors generation module is designed using correlation filtering which intrinsically belongs to a tracker. In each individual, the tracking of location and changes in scale is based on the observation that the positions of left ventricles in the images of the same individual are constrained in a similar location. In our future work, the proposed method will be extended to handle the structure of 3D CNN for detecting left ventricle and the tracking module will focus on the image volumes at different time points.

## 6. Conclusions

In this paper, in view of the large span of the left ventricle area in cardiac MRI, varying size of ventricular area, and the remarkable gray level and shape difference of the myocardial blood pool, a CNN detection method based on discriminative dictionary learning and sequence tracking is proposed for left ventricular localization. The left ventricle target region was fused by discriminating dictionary-classified superpixel region labels, and multi-scale adaptive anchors were generated in the target region. Combining with non-differential anchors in region proposal network, the left ventricle is detected by regression and classification strategy. To solve the problem of slow classification efficiency in discriminative dictionary learning, a sequence-tracking-based left ventricular scale adaptive anchors rapid generation module was proposed in the same individual. The proposed method and its variants were performed and compared to some classical algorithms in target detection on the public available heart atlas data set (CAP). The experimental results show the effectiveness of the proposed method in a series of evaluation indicators and that this method has the most competitive results on some evaluation indicators.

Considering the left ventricle in cardiac MRI is a three-dimensional tissue, our future work will focus on extending the proposed method to the three-dimensional detection of cardiac tissue and further estimate the posture of the heart in three-dimensional space.

## Figures and Tables

**Table 1:** Test results between the proposed method and the state-of-the-art methods (%).

Method	Backbone	P		R		F1		AP50	AP75
		IOU =	IOU =	IOU =	IOU =	IOU =	IOU =		
		0.5	0.75	0.5	0.75	0.5	0.75		
SSD	vgg-16	99	81.75	59.48	49.11	74.31	61.36	59.34	46
Retinanet	vgg-16	98.59	91.4	92.91	**86.14**	**95.67**	88.69	92.85	**85.39**
Yolov3	Darknet-53	98.7	86.3	77.21	67.52	86.64	75.76	64.12	77.07
Centernet	resnet18	**99.87**	**94.63**	86.09	81.58	92.47	87.62	86.08	80.87
Faster RCNN	vgg-16	97.37	90.62	91.01	84.7	94.08	87.56	90.88	83.95
Discriminant dictionary+Faster (Proposed)	vgg-16	98.58	90.9	91.92	84.77	95.13	87.73	91.86	84.17
Correlation filter+Faster (Proposed)	vgg-16	96.05	87.82	**93.09**	85.12	94.55	86.45	**92.95**	84.19
Correlation filter+Proposal (Proposed)	vgg-16	98.65	91.79	92.36	85.94	95.4	**88.77**	92.32	85.21

**Table 2:** Comparison of detection results by different algorithms in different individuals (%).

	AP50	AP75	AP50	AP75	AP50	AP75	AP50	AP75	AP50	AP75	AP50	AP75
	1		2		3		4		5		6	
SSD	52.17	39.25	59.34	52.66	26.87	22.04	64.68	48.86	41.89	23.43	63.51	38.89
Retinanet	92.33	84.66	94.81	**89.48**	91.04	**91.04**	85.71	84.42	**94.59**	**93.19**	**86.49**	82.03
Yolov3	69.5	63.06	89.61	77.6	76.47	57.76	84.4	78.65	83.5	70.62	69.8	64.57
Faster RCNN	92.39	82.96	92.21	83.08	88.06	83.58	84.42	84.42	91.89	83.54	82.43	68.96
Centernet	78.26	77.05	83.12	83.12	83.58	81.4	83.12	83.12	90.54	83.85	82.43	**82.43**
Discriminant dictionary + Faster (Proposed)	92.2	**86.81**	93.51	86.52	85.07	81.82	92.06	**88.18**	90.54	87.08	82.43	69.67
Correlation filter + Faster (Proposed)	**93.94**	77.4	94.81	85.57	**92.54**	86.34	**94.15**	87.9	93.2	86.06	82.43	69.9
Correlation filter + proposal (Proposed)	91.14	80.49	**96.09**	86.96	91.04	84.37	89.61	86.99	91.89	88.73	82.33	72.51
	**7**		**8**		**9**		**10**		**mAP50**	**mAP75**		
	**AP50**	**AP75**	**AP50**	**AP75**	**AP50**	**AP75**	**AP50**	**AP75**				
SSD	43.84	33.42	70.67	56.76	43.84	33.42	70.67	56.76	52.21	38.19		
Retinanet	79.45	71.89	**94.4**	**85.64**	79.45	71.89	**94.4**	**85.64**	90.05	**85.82**		
Yolov3	58.39	40.96	70.83	52.54	58.39	40.96	70.83	52.54	75.9	63.63		
Centernet	63.01	61.38	80.56	79.14	63.01	61.38	80.56	79.14	82.09	80.35		
Faster RCNN	68.36	65.56	81.94	69.6	68.36	65.56	81.94	69.6	86.35	79.32		
Discriminant dictionary + Faster (Proposed)	75.34	**72.42**	84.29	78.55	75.34	**72.42**	84.29	78.55	87.58	81.57		
Correlation filter + Faster (Proposed)	77.86	70.9	92.89	80.24	77.86	70.9	92.89	80.24	**90.5**	81.67		
Correlation filter + proposal (Proposed)	**81.93**	72.05	87.46	84.66	**81.93**	72.05	87.46	84.66	89.32	82.96		

**Table 3:** Comparison of detection time between the proposed method in this paper and a series of detection algorithms (s/image), where D-d+Faster, C-f+Faster, C-f+Proposal denotes the proposed Discriminant dictionary + Faster, and Correlation filter + Faster, Correlation filter + Proposal methods, respectively.

	SSD	Yolov3	Retinanet	Centernet
Test time	0.198	**0.023**	0.052	0.046
	**Faster RCNN**	**D-d + Faster**	**C-f + Faster**	**C-f + Proposal**
Test time	0.311	8.31	0.339	0.287

## Data Availability

The data presented in this study are publicly available. Refer to the description of each data set.
